# Ultrasound-Mediated Cavitation Enhances EGFR-Targeting PLGA-PEG Nano-Micelle Delivery for Triple-Negative Breast Cancer Treatment

**DOI:** 10.3390/cancers13143383

**Published:** 2021-07-06

**Authors:** Libin Chen, Tao Zhang, Shan Sun, Wenzhi Ren, Aiguo Wu, Huixiong Xu

**Affiliations:** 1 School of Medicine, Tongji University, Shanghai 200072, China; 1811233@tongji.edu.cn; 2 Department of Ultrasound in Medicine, Ningbo First Hospital, Ningbo 315010, China; 3 Department of Ultrasound in Medicine, The Second Affiliated Hospital of Zhejiang University School of Medicine, Hangzhou 310009, China; zhangtao-us@zju.edu.cn; 4 Cixi Institute of Biomedical Engineering, International Cooperation Base of Biomedical Materials Technology and Application, CAS Key Laboratory of Magnetic Materials and Devices & Zhejiang Engineering Research Center for Biomedical Materials, Ningbo Institute of Materials Technology and Engineering, Chinese Academy of Sciences, 1219 ZhongGuan West Road, Ningbo 315201, China; sunshan@nimte.ac.cn (S.S.); renwzh@nimte.ac.cn (W.R.); 5 Center of Minimally Invasive Treatment for Tumor, Department of Medical Ultrasound, Shanghai Tenth People’s Hospital, Ultrasound Research and Education Institute, Clinical Research Center for Interventional Medicine, School of Medicine, Tongji University, Shanghai Engineering Research Center of Ultrasound Diagnosis and Treatment, National Clinical Research Center for Interventional Medicine, 301 Yanchangzhong Rd, Shanghai 200072, China

**Keywords:** ultrasound-mediated cavitation, EGFR-targeting, triple-negative breast cancer, DOX, PLGA nano-micelle, SonoVue^TM^

## Abstract

**Simple Summary:**

Triple-negative breast cancer (TNBC) with negative expression of estrogen receptor (ER), progesterone receptor (PR), and human epidermal growth factor 2 (HER2) is considered to be associated with poorer outcomes and a higher risk of recurrence or metastasis owing to a lack of effective targeted therapeutic drugs. The epidermal growth factor receptor (EGFR) functions is a driver of disease progression in most of TNBC that represents a viable target that can be leveraged to guide the intra-tumoral delivery of chemotherapeutic drugs in TNBC patients. Moreover, ultrasound-mediated cavitation (UMC) strategies increase tissue permeability and extravasation through nuclei-dependent cavitation via sonoporation, thus enabling drugs to better enter target tissues. In this research, a combination of active, targeting nano-micelles and UMC was able to inhibit TNBC tumor growth effectively at lower concentrations while reducing treatment-related toxicity. Thus, this is a very promising treatment strategy in the clinical therapy with TNBC and other cancer types.

**Abstract:**

Triple-negative breast cancer (TNBC) is highly recurring and metastatic breast cancer with overexpressing epidermal growth factor receptor (EGFR). Herein, a series of in vitro and in vivo analyses were used to explore the therapeutic effect of EGFR-targeting nano-micelles (PLGA-PEG/DOX@anti-EGFR) combined with ultrasound-mediated cavitation (UMC). The prepared nano-micelle drug carriers have good biocompatibility and can greatly increase the drug accumulation in tumor regions, thereby reducing off-target toxicity while enhancing anti-tumor efficacy. Moreover, an in vivo analysis of the practical utility of this treatment modality was conducted by using SonoVue^TM^ microbubbles to achieve cavitation under different power intensity levels, with an ultrasonic power intensity of 0.5 W/cm^2^ maximizing the intra-tumoral blood perfusion. Relative to PLGA-PEG@DOX/anti-EGFR nano-micelles treatment alone, the combination with UMC was better able to suppress tumor growth even at low concentrations. As such, combining actively targeted drug-carrier molecules with UMC represents an effective approach to enhancing therapeutic efficacy while reducing the adverse, systemic effects associated with DOX and other chemotherapeutic drugs, and it can be considered as a promising clinical prospect in the treatment of TNBC.

## 1. Introduction

Triple-negative breast cancer (TNBC) refers to a form of breast malignancy that is negative for the expression of estrogen receptor (ER), progesterone receptor (PR), and human epidermal growth factor 2 (HER2), accounting for 10–15% of human breast cancers [1,2]. TNBC is generally considered to be associated with poorer prognosis and a higher risk of recurrence or metastasis owing to a lack of effective targeted therapeutic drugs [3,4,5]. At present, a combination of surgical resection and adjuvant chemotherapy/radiotherapy is the standard treatment for individuals with TNBC. More than half of TNBC patients exhibit the overexpression of epidermal growth factor receptor (EGFR) in tumor cells, and such overexpression is correlated with a poorer prognosis [6]. Some studies have suggested that EGFR may function as a driver of disease progression in most cases of TNBC [7,8]. As such, EGFR may represent a viable target that can be leveraged to guide the intra-tumoral delivery of chemotherapeutic agents or other drugs in TNBC patients. While researchers have sought to disrupt TNBC tumor growth using EGFR-blocking antibodies or other approaches for EGFR degradation, these treatments have failed to achieve beneficial outcomes in many TNBC patients [3,9,10], instead exhibiting a high degree of response heterogeneity. As such, there is a clear need for the development of novel therapeutic strategies capable of enhancing the chemo-therapeutic effect, reducing off-target toxicity, and delaying progression.

Doxorubicin (DOX) is an anthracycline chemotherapeutic drug that is widely used to efficiently treat diverse tumor types, including breast cancer, lung cancer, and lymphoma [11]. While it can exhibit robust antitumor activity, the clinical efficacy of DOX is constrained by its toxicity profile, particularly owing to its ability to induce delayed cumulative cardiotoxicity. In an effort to reduce these toxic side effects and enhance the clinical utility of DOX, many have sought to develop nano-scale drug-delivery systems [12], with lipid-based particles being the most common particle type owing to their perceived safety. Nanomedicines offer a number of advantages over traditional drug-delivery strategies, including excellent bioavailability, prolonged stability while in circulation, improved drug solubility, an ability to shield off-target tissues from the effects of the encapsulated drugs [13,14,15,16], and a lower risk of opsonization [17,18]. The enhanced permeability and retention effect (EPR) enables the preferential accumulation of nanoparticles within tumor tissues [19] owing to the relatively poor lymphatic drainage and dysregulated endothelial vasculature within these malignancies [20]. However, one recent meta-analysis of preclinical studies conducted over the past decade found that, on average, just 0.7% of injected nanoparticles ultimately undergo intra-tumoral deposition [21], likely owing to the series of physiological barriers to tumor-cell entry [22]. Actively targeted nanoparticles thus represent a more promising approach to cancer treatment, with such targeting being mediated by the conjugation of specific antibodies or other molecules at the outer nanoparticle surface. By leveraging specific receptor-ligand or antibody binding interactions, it is possible to ensure that these nanoparticles accumulate more readily in tumors than in healthy tissues.

Besides the active targeting strategy, local intra-tumoral drug accumulation can also be enhanced by increasing the permeability of vascular and other physiological barriers via ultrasound-mediated cavitation (UMC). UMC strategy can increase tissue permeability and extravasation through sonoporation-induced cavitation, thus enabling drugs to better enter target tissues [23,24,25,26,27]. These ultrasound-based treatment approaches are not restricted by depth and have been shown to exhibit therapeutic benefits in pancreatic cancer patients with inoperable disease [28,29]. 

**Scheme 1:**
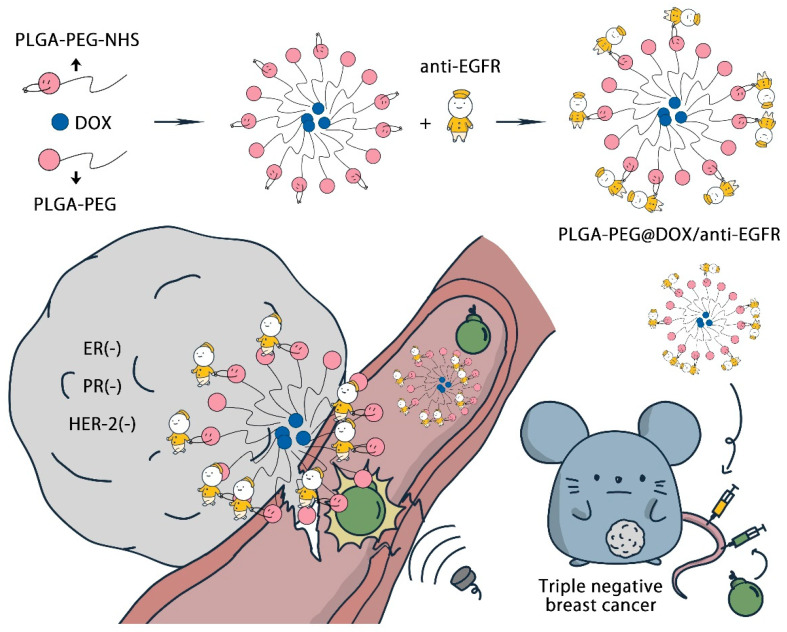
The synthesis of PLGA-PEG@DOX/anti-EGFR nano-micelles and schematic illustration of their subsequent application in UMC combined with chemotherapy.

The present study was designed to assess the ability of DOX-loaded nano-micelles to treat TNBC tumors with or without UMC and to assess the ability of anti-EGFR targeting to enhance the efficacy of this therapeutic approach. PLGA-PEG/DOX@anti-EGFR nano-micelles were thus synthesized and applied in combination with UMC to treat TNBC in vitro or in vivo (Scheme 1). These PLGA-PEG/DOX@anti-EGFR nano-micelles were found to significantly improve TNBC treatment outcomes, inhibiting tumor growth while reducing treatment-related toxicity owing to the enhanced EGFR-mediated targeting and UMC-induced vascular permeability. 

## 2. Materials and Methods

### 2.1. Materials

Poly (DL-lactide-co-glycolide)-b-Poly (ethylene glycol) (PLGA_2k_-PEG_2k_) and Poly (DL-lactide-co-glycolide)-b-Poly (ethylene glycol)-NHS (PLGA_2k_-PEG_2k_-NHS) were from Shanghai Yare Biotech Company (Shanghai, China). Anti-EGFR was from MedChemExpress (Princeton, NJ, USA). A Calcein-AM/P staining kit was purchased from Yeasen Biological Technology (Shanghai, China). Millipore Milli-Q grade (18.2 MΩ) dH_2_O was utilized for all experiments, and all chemicals were utilized without additional purification.

### 2.2. Animals and Cell Lines

MDA-MB-468 cells were purchased from Shanghai Cell Bank and cultured in RPMI-1640 (Hyclone, Logan, UT, USA) containing 10% FBS (Hyclone, Logan, UT, USA) at 37 °C in a 5% CO_2_ incubator. Nude mice (5–6 weeks old) were obtained from BiKai Biological products sales center (Nanjing, China). The Ningbo University’s Regional Ethics Committee for Animal Experiments approved all animal studies described herein (Permit No. SYXK (Zhe) 2019-0005).

### 2.3. PLGA-PEG/DOX@anti-EGFR Synthesis

PLGA-PEG/DOX nano-micelles were prepared by initially combining 10 mL of acetone with 10 mg of doxorubicin hydrochloride and 100 µL of triethylamine and mixing for 24 h at room temperature. A 1 mL volume of the supernatant from this solution was then mixed with 2 mL of acetone containing 25 mg PLGA-PEG, followed by a 5 min sonication step. This solution was then added dropwise to 2 mL of dH_2_O and was stirred for 5 min. A roto-evaporator (Hangzhou GengYu Instrument Co., Limited, Hangzhou, China) was then utilized to remove acetone from this solution at 150 rpm at 45 °C. Following evaporation for 30 min, solutions were passed through a 0.22 µm Millipore filter, yielding approximately 1.2 mL of a PLGA-PEG/DOX solution. To prepare PLGA-PEG/DOX@anti-EGFR nano-micelles, the steps were identical to those above except that 2.5 mg of PLGA-PEG was substituted for an equal amount of PLGA-PEG-NHS, thus yielding a PLGA-PEG(-NHS)/DOX solution that was subsequently mixed for 24 h at room temperature with anti-EGFR (0.2 mg) at room temperature. The solution was then passed through an Ultrafiltration tube (Millipore 100 kDa) at 15,000 g for 10 min, with the resultant supernatant being isolated as a PLGA-PEG/DOX@anti-EGFR solution.

### 2.4. Instrumentation

A particle analyzer (Nano-ZS, Malvern Panalytical Ltd, Malvern, England) was used to assess nano-micelle DLS and zeta potential properties at room temperature. High-resolution transmission electron microscopy (HRTEM) images were captured with a JEOL2100 instrument (JEOL, Ltd., Tokyo, Japan). UV–vis spectroscopy (Lambda 950, PerkinElmer, Shelton, CT, USA) was used to assess DOX encapsulation within prepared nano-micelles. A flow cytometer (BD FACSCantoTM II, BD Biosciences, San Jose, CA, USA) was used for appropriate analyses. Nano-micelle distribution patterns in vivo were assessed via confocal laser scanning microscopy (FV1200, Olympus, Tokyo, Japan) and with an IVIS Lumina XRMS Series III instrument (PerkinElmer). Ultrasound cavitation was used by physiotherapy ultrasound device (US-750, ITO Physiotherapy & Rehabilitation Co., LTD., Tokyo, Japan). The contrast enhanced ultrasound (CEUS) was performed with Resona 7 Ultrasound system (Mindray, Shenzhen, China). 

### 2.5. pH-Dependent Drug Release Analysis

Cumulative DOX release from PLGA-PEG/DOX@anti-EGFR nano-micelles was assessed via a dynamic dialysis approach [30]. Briefly, a 1 mL volume of PLGA-PEG/DOX@anti-EGFR (1 mg/mL) was added to a dialysis bag in 20 mL of PBS (pH = 6.3 or 7.4) (MWCO: 1 kDa), followed by dialysis at 100 rpm in an oscillation incubator. At appropriate time points, 1 mL samples were removed and replaced with an equivalent volume of appropriate PBS solutions. DOX release was then assessed with a UV-Vis spectrophotometer. 

### 2.6. Cell Uptake Studies 

A confocal laser scanning microscopy (CLSM) approach was used to assess cellular PLGA-PEG/DOX and PLGA-PEG/DOX@anti-EGFR uptake. Briefly, MDA-MB-468 cells (1 × 10^5^) were added to confocal culture dishes for 24 h, after which PLGA-PEG/DOX or PLGA-PEG/DOX@anti-EGFR (100 µg/mL) were added. Following an 8 h incubation, plates were washed thrice with PBS, stained with Hoechst 33258 (10 µg/mL) for 30 min at room temperature, and analyzed via CLSM. Flow cytometry was additionally used to assess nano-micelle uptake. Briefly, cells were plated as above and then treated with PLGA-PEG/DOX or PLGA-PEG/DOX@anti-EGFR nano-micelles (5 µg/mL DOX equivalent dose) for 2, 4, 8, or 12 h. Samples were then washed with PBS and assessed using a flow cytometer (λ_ex_ = 480 nm, λ_em_ = 570 nm).

### 2.7. Transwell Vascular Permeability Assay

MDA-MB-468 cells were added to 96-well plates (5 × 10^4^/mL, 100 µL/well) for 24 h. Vascular permeability was then assessed using Transwell inserts (polycarbonate filters, 0.4 µm pore size; Corning Costar, Corning, NY, USA). Briefly, an endothelial cell monolayer was cultured on this insert prior to exposure to US irradiation (0.5 w/cm^2^, duty cycle: 50%, 1 min) following the addition of 10 µL of SonoVue. Following treatment, 100 µL of sample from the lower compartment was removed, placed in a 96-well plate, and incubated for 24 h. Viability was then assessed via microplate reader at 450 nm. 

### 2.8. In Vitro Cytotoxicity Analysis

MDA-MB-468 cells were added to 96-well plates (5 × 10^4^/mL, 100 µL/well) for 24 h, after which media was exchanged for 100 µL of media containing free DOX, PLGA-PEG/DOX, or PLGA-PEG/DOX@anti-EGFR (at DOX equivalent doses of 0, 2.5, 5, 10, 20, or 40 µg/mL). UMC treatment either was or was not performed. Following an additional 24 h incubation, viability was assessed at 450 nm via microplate reader. 

### 2.9. Live/Dead Staining

A Calcein-AM/PI staining kit was employed to assess tumor-cell viability. Briefly, MDA-MB-468 cells were added to 96-well plates (5 × 10^4^/mL, 100 µL/well) for 24 h, after which media was exchanged for 100 µL of media containing free DOX, PLGA-PEG/DOX, or PLGA-PEG/DOX@anti-EGFR (10 µg/mL DOX equivalent dose). After 24 h, cells were stained for 30 min with a Calcein-AM/PI solution at 37 °C. Samples were then imaged via fluorescence microscopy.

### 2.10. Biocompatibility Testing

Healthy nude mice were intravenously administered 100 µL of PBS or PLGA-PEG@anti-EGFR (5.0 mg/mL) and were monitored for a 21-day period for any behavioral changes. After this, animals were euthanized, and samples of blood and major organs were collected and subjected to routine analyses, with tissue samples being fixed with 4% paraformaldehyde, H&E stained, and examined to assess PLGA-PEG@anti-EGFR toxicity.

### 2.11. B-Mode and CEUS Perfusion Imaging

B-mode and contrast-enhanced ultrasound (CEUS) imaging were performed with an L5-14 linear scanner in the 5.0–14.0 MHz frequency range (Resona 7, Mindray, Shenzhen, China). Phospholipid-coated sulfur hexafluoride microbubbles (SonoVue^TM^, Bracco, Italy) were utilized for contrast, with concentrations of 1–5 × 10^8^ microbubbles/mL and a mean diameter of 2.5 µm [31]. Briefly, 100 µL of the stock microbubble solution was diluted in 1 mL of saline solution, after which 100 µL of this solution was administered intravenously to mice to facilitate CEUS imaging. Fixed depth, gain, and other settings were used in all CEUS imaging analyses, and resultant peak intensity (PI) values were determined with the provided built-in quantification software (Resona 7, Mindray, Shenzhen, China). In B-mode tumor images, a region of interest (ROI) incorporating the entire tumor was defined. 

### 2.12. In Vivo Animal Treatment

UMC treatments were conducted with a physiotherapy ultrasound device (US-750, ITO physiotherapy and rehabilitation Co., LTD., Tokyo, Japan). Vascular permeability was calculated based upon peak intensity (PI) values derived from CEUS experiments. UMC parameter optimization of variables associated with increased vascular permeability including power density, duty cycle, irradiation time, and SonoVue^TM^ dosage was initially performed prior to in vivo experimentation.

For mouse-model experiments, tumor-bearing animals were injected with 1 × 10^7^ tumor cells in the right fat pad of the third pair of breasts. Once tumors were 40–60 mm^3^ in size, mice were randomly assigned to eight groups (*n* = 5/group): PBS, PBS + UMC, free DOX, DOX + UMC, PLGA-PEG@DOX, PLGA-PEG@DOX + UMC, PLGA-PEG@DOX/anti-EGFR, and PLGA-PEG@DOX/anti-EGFR + UMC groups. Appropriate treatments were administered intravenously in a 100 µL volume (PBS, free DOX, PLGA-PEG@DOX, or PLGA-PEG@DOX/anti-EGFR; 5 mg/kg DOX equivalent dose) with or without US irradiation (0.5 W/cm^2^, duty cycle: 50%, 5 min) as appropriate. The whole process was repeated on a weekly basis. Tumors were measured every three days with calipers, after which tumor volume was determined as follows: tumor volume (mm^3^) = length × width^2^/2. Body weight was measured every other day. On day 15 post-treatment, mice were euthanized, and tumors were resected for analysis. 

### 2.13. Assessment of DOX Tumor Tissue Penetration 

DOX and PLGA-PEG@DOX/anti-EGFR (5 mg/kg for DOX) were intravenously injected into mice bearing MDA-MB-468 tumors. At 24 h post-injection, tumors were isolated and frozen in OCT medium (Sakura Finetek, Torrance, CA, USA) at −80 °C. Tumors were then cut to yield 6 µm sections, dried for 10 min, and fixed for 10 min with 4% paraformaldehyde (Solarbio, Beijing, China). DAPI (Invitrogen, Carlsbad, CA, USA) was then used for nuclear counterstaining, with anti-CD31 (Abcam, Cambridge, UK) being used to stain blood vessels within tumors, after which samples were assessed via confocal microscope. 

### 2.14. Pharmacokinetics Analyses 

Female nude mice (7–8 weeks old) were intravenously administered DOX or PLGA-PEG@DOX/anti-EGFR (5 mg/kg for DOX, *n* = 3/group). Samples of blood were collected retro-orbitally at 3 min, 20 min, 1 h, 2 h, 4 h, 8 h, 12 h, and 24 h post-injection in tubes containing EDTA anticoagulant (BD, Franklin Lakes, NJ, USA). Samples were spun for 10 min at 4500 rpm, after which supernatants were combined with an equal volume of acetonitrile to facilitate plasma protein precipitation. Samples were again centrifuged, after which supernatants were collected for UV-vis spectroscopy analyses. 

## 3. Results and Discussion

### 3.1. PLGA-PEG@DOX/anti-EGFR Nano-Micelle Characterization

**Figure 1:**
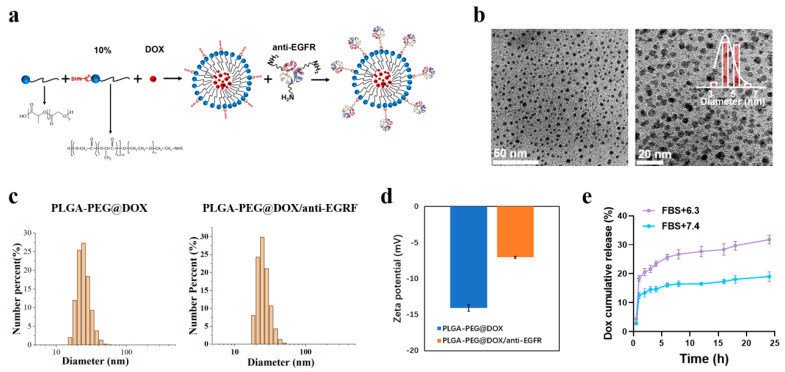
(**a**) PLGA-PEG@DOX/anti-EGFR nano-micelle synthesis strategy. (**b**) PLGA-PEG@DOX/anti-EGFR nano-micelles were assessed via TEM. (**c**) DLS was used to assess PLGA-PEG@DOX and PLGA-PEG@DOX/anti-EGFR nano-micelle size distributions. (**d**) PLGA-PEG@DOX and PLGA-PEG@DOX/anti-EGFR nano-micelle zeta potential values. (**e**) Cumulative DOX release from PLGA-PEG@DOX/anti-EGFR (pH = 7.4 or 6.3) in vitro over the course of 24 h in a 10% FBS solution at 37 °C. Samples were assessed in triplicate.

After successfully synthesizing PLGA-PEG/DOX and PLGA-PEG/DOX@anti-EGFR nano-micelles (Figure 1a), transmission electron microscopy (TEM) analyses indicated that these nano-micelles were 4.78 ± 0.53 nm and 4.91 ± 0.60 nm in size, respectively (Figure 1b and Figure S1). The hydrodynamic sizes of these PLGA-PEG/DOX and PLGA-PEG/DOX@anti-EGFR nano-micelles were then measured via dynamic light scattering (DLS) as 28.39 ± 0.46 nm (PDI = 0.154) and 28.56 ± 0.17 nm (PDI = 0.106), respectively (Figure 1c). These results confirmed that our nano-micelles exhibited uniform size distributions, with the larger diameter values in DLS analyses being attributable to superhydrophilic phosphorylcholine and water layers associated with these nanoparticles [32]. The PLGA-PEG/DOX and PLGA-PEG/DOX@anti-EGFR nano-micelles exhibited respective zeta potential values of −14.07 ± 0.45 mV and −7.05 ± 0.13 mV at a pH of 7.4 (Figure 1d). Importantly, prepared PLGA-PEG/DOX@anti-EGFR nano-micelles remained stable without any significant changes in diameter over a 3-day period in cell-culture media at 37 °C (Figure S2). DOX encapsulation efficiency in these nano-micelles was additionally assessed using standard curves (Figure S3), with a calculated encapsulation efficiency value of 85.17%. Samples were then incubated at a pH of 6.3 or 7.4 in a 10% FBS solution at 37 °C for 24 h, after which cumulative DOX release levels were 31.77 ± 1.25% and 19.02 ± 1.37%, respectively (Figure 1e), suggesting that DOX is more readily released under acidic conditions.

### 3.2. Ultrasonic Cavitation Induces Increased Vascularpermeability

**Figure 2:**
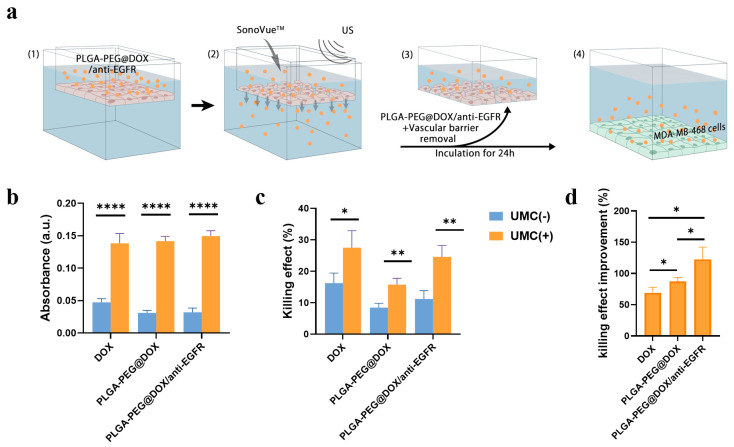
Ultrasonic cavitation-induced vascular permeability enhances DOX delivery to tumor cells. (**a**) Experimental overview: (1) free DOX, PLGA-PEG@DOX, or PLGA-PEG@DOX/anti-EGFR nano-micelles were added, after which (2) UMC treatment of the vascular barrier was performed, and (3) excess drug or nano-micelles were removed, after which the levels of these agents that had penetrated the vascular barrier was assessed, with (4) a CCK-8 assay being used to examine the impact of PBS, free DOX, PLGA-PEG@DOX, and PLGA-PEG@DOX/anti-EGFR nano-micelles on MDA-MB-468 cell viability. (**b**) UMC treatment promoted the penetration of free DOX, PLGA-PEG@DOX, and PLGA-PEG@DOX/anti-EGFR nano-micelles across the vascular barrier and (**c**,**d**) thereby significantly enhanced the cytotoxicity of these treatments towards MDA-MB-468 tumor cells owing to their ability to more efficiently cross the vascular layer (*n* = 3). * *p* < 0.05, ** *p* < 0.01, **** *p* < 0.0001. UMC(+) = with ultrasound-mediated cavitation, UMC(−) = without ultrasound-mediated cavitation.

To examine the therapeutic efficacy of UMC treatment as a means of enhancing vascular permeability, a proof-of-concept assay was conducted (Figure 2a). Briefly, a simulated vascular barrier was formed, after which it was treated with SonoVue^TM^ (10 µL/mL) to generate a model of vascular leakage, followed by free DOX, PLGA-PEG@DOX, or PLGA-PEG@DOX/anti-EGFR nano-micelle treatment and assessment of the effects of these compounds on MDA-MB-468 cells. The safety under various power intensity (0–1.5 W/cm^2^) was analyzed, which showed no obvious toxicity to the cells (Figure S4). While only small amounts of DOX were able to pass through the untreated vascular barrier, UMC pretreatment markedly enhanced barrier permeability (Figure 2b). We further found that the cytotoxic efficacy of these treatments was enhanced by UMC pretreatment, with respective improvements in killing efficacy of 69.10%, 87.08%, and 122.53% in the DOX, PLGA-PEG@DOX, and PLGA-PEG@DOX/anti-EGFR nano-micelle treatment groups (Figure 2c,d). These analyses confirmed that ultrasonic cavitation played a central role in the induction of vascular permeability. 

### 3.3. Analysis of Nano-Micelle Cellular Uptake and In Vitro Therapeutic Utility

**Figure 3:**
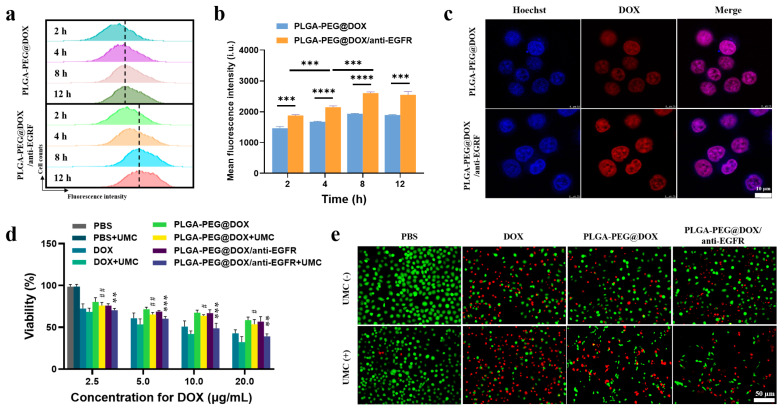
Flow cytometry (**a**) and corresponding mean fluorescence intensity values (**b**) for MDA-MB-468 cells following treatment with PLGA-PEG@DOX or PLGA-PEG@DOX/anti-EGFR nano-micelles (5 µg/mL DOX equivalent dose) for the indicated amounts of time (*n* = 3). *** *p* < 0.001, **** *p* < 0.0001. (**c**) MDA-MB-468 cells were assessed via confocal microscope following an 8 h treatment with PLGA-PEG@DOX/anti-EGFR nano-micelles (5 µg/mL DOX equivalent dose). Scale bar: 10 µm. (**d**) MDA-MB-468 cell viability was assessed following the indicated treatments with a range of DOX doses (2.5–20 µg/mL) for 24 h. Data are means ± standard deviation (*n* = 5). ^#^ PLGA-PEG@DOX+UMC vs. PLGA-PEG@DOX/anti-EGFR+UMC. ^#^ *p* < 0.05, ^##^ *p* < 0.01. * PLGA-PEG@DOX/anti-EGFR vs. PLGA-PEG@DOX/anti-EGFR+UMC. ** *p* < 0.01, *** *p* < 0.001. (**e**) MDA-MB-468 cells fluorescence was assessed following the indicated treatment. Scale bar: 50 µm. UMC(+) = with ultrasound-mediated cavitation, UMC(−) = without ultrasound-mediated cavitation.

Flow cytometry and CLSM were used to assess the uptake of PLGA-PEG@DOX/anti-EGFR by human MDA-MB-468 TNBC cells overexpressing EGFR. Mean fluorescence intensity (MFI) values for these cells were significantly higher following PLGA-PEG@DOX/anti-EGFR nano-micelle treatment relative to PLGA-PEG@DOX treatment (Figure 3a,b) (*p* < 0.001), confirming that this targeting mechanism enhanced treatment efficacy. The uptake of both PLGA-PEG@DOX and PLGA-PEG@DOX/anti-EGFR nano-micelles was time-dependent; the uptake plateau was observed at 8 h. As such, subsequent assays were conducted with an 8 h incubation period. CLSM further confirmed the active targeting activity of PLGA-PEG@DOX/anti-EGFR treatment in MDA-MB-468 cells, with a more robust DOX fluorescent signal in the nuclei of MDA-MB-468 cells following PLGA-PEG@DOX/anti-EGFR treatment relative to PLGA-PEG@DOX treatment at 8 h post-nano-micelle administration (Figure 3c). Human breast cancer cell lines with high and low EGFR expression (MDA-MB-468 and MCF-7, respectively) were used to study the selectivity of nano-micelles uptake. As shown in Figure S5, the uptake of anti-EGFR targeting nano-micelles in MBA-DA-468 cells was significantly increased, while the changes in the uptake of MCF-7 cell lines are not obvious, indicating that PLGA-PEG@DOX/anti-EGFR nano-micelle can increase the uptake of TNBC through active targeting effect.

A CCK-8 assay was next used to compare the ability of free DOX and our different nano-micelle preparations to suppress MDA-MB-468cell growth in the presence or absence of UMC treatment (Figure 3d). These analyses revealed that nano-micelles suppressed tumor growth in a dose-dependent manner, with combination UMC + nano-micelle treatment being linked to increased tumor-cell death as compared to UMC or nano-micelle treatment in isolation. These data confirmed the ability of UMC treatment to enhance permeability and hereby augment chemotherapeutic drug efficacy. These therapeutic effects were further enhanced in the context of EGFR targeting. When a Calcein-AM/PI staining kit was further used to assess cellular viability (Figure 3e), it was confirmed that combined nano-micelle + UMC treatment was similarly linked to more efficient tumor cell destruction relative to either of these treatments in isolation.

### 3.4. In Vivo Assessment of UMC Enhancement of TNBC Tumor Perfusion

**Figure 4:**
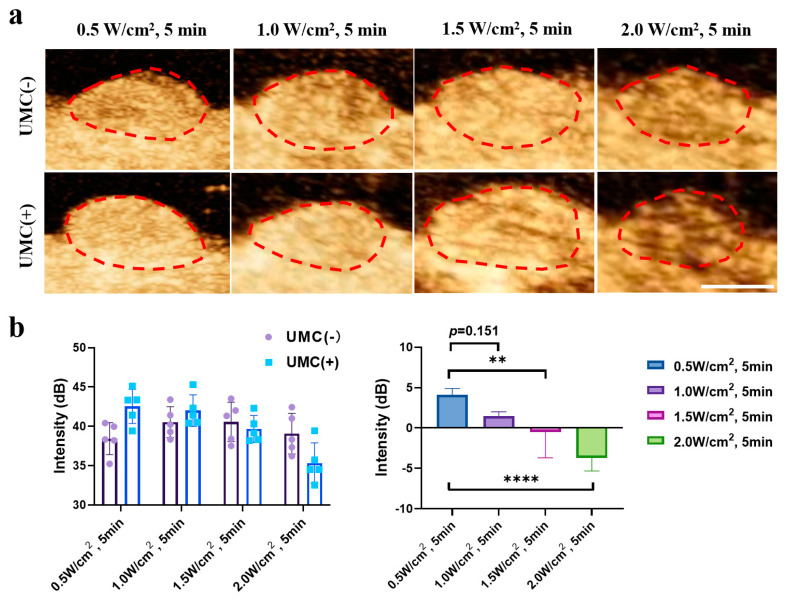
Assessment of UMC-induced changes in tumor perfusion. (**a**) The intra-tumoral perfusion of MDA-MB-468 tumor-bearing mice was assessed via CEUS pre- and post-treatment. (**b**) PI values from pre- and post-treatment CEUS analyses. Data are means ± standard deviation (*n* = 5). ** *p* < 0.01, **** *p* < 0.0001. Scale bar: 5 mm. UMC(+) = with ultrasound-mediated cavitation, UMC(−) = without ultrasound-mediated cavitation.

UMC can disrupt vascular wall integrity, thereby enhancing permeability and augmenting blood perfusion within tumor cells. Contrast enhanced ultrasound (CEUS) is a non-invasive and reliable imaging method for determining tumor perfusion [33]. When CEUS was used to assess pre- and post-treatment tumor perfusion, power density levels of 0.5 and 1.0 W/cm^2^ were found to significantly enhance such perfusion with corresponding increases in PI and AUC values, whereas higher 1.5 and 2.0 W/cm^2^ power density levels reduced such perfusion and PI values (Figure 4a). PI values differed significantly before and after CEUS (Figure 4b).

### 3.5. In Vivo Biocompatibility and Distribution Analyses

The biocompatibility of prepared PLGA-PEG/anti-EGFR nano-micelles was next assessed by injecting them into healthy nude mice. Within the 21-day period following injection, no weight loss (Figure S6) or behavioral changes were observed relative to control animals, and no changes were detected in routine hematological analyses (Figure S7), analyses of kidney and liver function (Figure S8), or H&E staining of primary organs (Figure S9). Together, these results suggest that these PLGA-PEG/anti-EGFR nano-carriers were not associated with any intrinsic, treatment-related, toxic side effects. 

**Figure 5:**
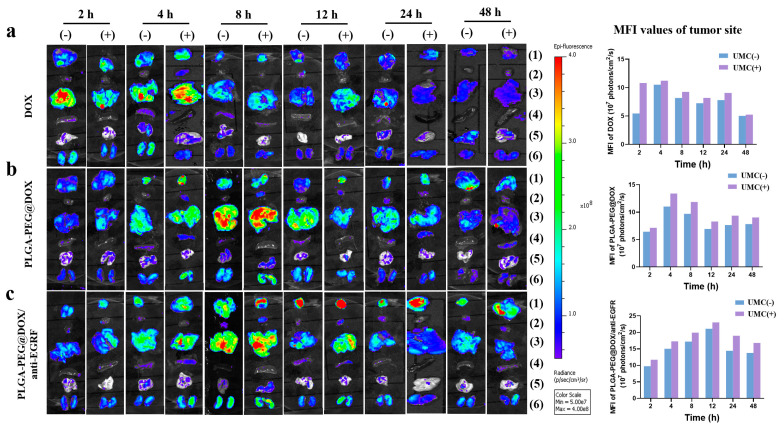
Biodistribution studies of free DOX, PLGA-PEG@DOX, and PLGA-PEG@DOX/anti-EGFR nano-micelles. MBA-MD-468 tumor-bearing mice were intravenously administered appropriate nano-micelles or free drug. Ex vivo fluorescence images of tumor (1) and major organs, including the heart (2), liver (3), spleen (4), lungs (5), and kidneys (6), in these mice were assessed at 2, 4, 8, 12, 24, and 48 h following free DOX (**a**), PLGA-PEG@DOX (**b**), or PLGA-PEG@DOX/anti-EGFR nano-micelle (**c**) administration. UMC(+) = with ultrasound-mediated cavitation, UMC(−) = without ultrasound-mediated cavitation.

To evaluate the biodistribution of our prepared nano-micelles, we conducted ex vivo imaging of tumor and major organs in MBA-MD-468 tumor-bearing mice that had been administered free DOX, PLGA-PEG@DOX, or PLGA-PEG@DOX/anti-EGFR nano-micelles at 2, 4, 8, 12, 24, and 48 h post-treatment. Relative to mice administered free DOX that were intravenously injected with PLGA-PEG@DOX and PLGA-PEG@DOX/anti-EGFR nano-micelles (Figure 5), they exhibited significantly enhanced intra-tumoral DOX accumulation, with the active targeting activity of PLGA-PEG@DOX/anti-EGFR nano-micelles being linked to the most effective DOX accumulation. UMC treatment was associated with significantly enhanced intra-tumoral fluorescence relative to that observed in mice not subjected to UMC treatment at all time points. A combination of active targeting mechanisms and UMC treatment can enhance in vivo drug delivery to TNBC tumors.

### 3.6. In Vivo Tumor Inhibition 

**Figure 6:**
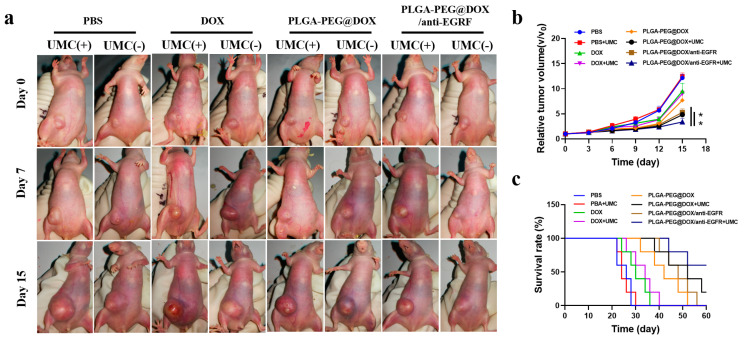
(**a**) Representative images of MBA-MD-468 tumor-bearing mice in the indicated treatment groups. (**b**) Relative tumor volume and (**c**) murine survival rate over time were assessed in the indicated treatment groups. Data are means ± standard deviation (*n* = 5). ** *p* < 0.01. UMC(+) = with ultrasound-mediated cavitation, UMC(−) = without ultrasound-mediated cavitation.

The in vivo antitumor activity was assessed by intravenously administering PBS, free DOX, PLGA-PEG@DOX, or PLGA-PEG@DOX/anti-EGFR nano-micelles in animals harboring tumors that were 40–60 mm^3^ in size, followed by UMC treatment in appropriate groups. As shown in Figure 6a,b, the tumor-growth inhibition value (TGI) of mice was most significant in the PLGA-PEG@DOX/anti-EGFR + UMC group (72.14 ± 5.36%), followed by the PLGA-PEG@DOX + UMC (60.31 ± 3.47%) and PLGA-PEG@DOX/anti-EGFR group(56.49 ± 5.09%). While control mice survived for up to 28 days, mice treated with PEG@DOX/anti-EGFR + UMC survived for as many as 60 days (Figure 6c). As such, UMC treatment and EGFR targeting are effective in promoting nano-micelle-mediated intra-tumoral drug delivery. Increased tumor volumes usually necessitate treatment with higher doses of chemotherapeutic drugs, resulting in pronounced side effects, including substantial cardiotoxicity [34]. The ability of our combined nano-micelle and UMC therapeutic strategy to enhance the amount of DOX within tumors without increasing the overall amount of this compound that was administered has the potential to be of key clinical benefit. Moreover, there were no obvious weight losses in all groups (Figure S10), indicating that the systemic toxicity of PLGA-PEG@DOX/anti-EGFR nano-micelles during treatment is negligible.

**Figure 7:**
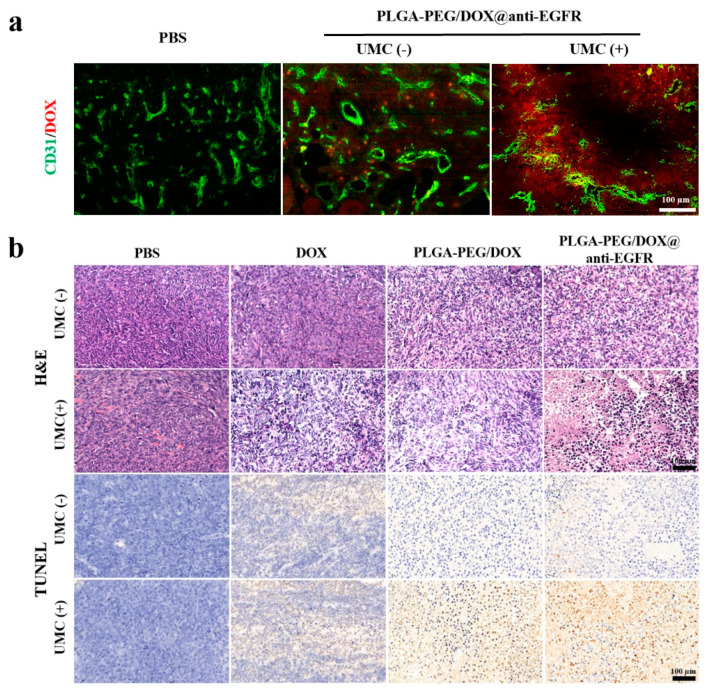
(**a**) DOX intra-tumoral penetration following intravenous PLGA-PEG@DOX/anti-EGFR injection with or without UMC. (**b**) H&E and TUNEL staining of MBA-MD-468 tumor samples in the indicated treatment groups. Scale bar: 100 µm. UMC(+) = with ultrasound-mediated cavitation, UMC(−) = without ultrasound-mediated cavitation.

PLGA-PEG@DOX/anti-EGFR intra-tumoral drug delivery in vivo was assessed in combination with or without UMC treatment. Mice were intravenously administered PLGA-PEG@DOX/anti-EGFR (5 mg/kg DOX equivalent dose) or an equivalent volume of PBS, and tumor tissues were isolated at 24 h post-treatment. Confocal imaging of these tumors was then performed, with CD31 being used to stain the tumor-associated vasculature, revealing a gradient of DOX-associated fluorescence extending from the edge to the core of the tumor, with this fluorescence being more robust in the PLGA-PEG@DOX/anti-EGFR + UMC treatment group relative to the control and PLGA-PEG@DOX/anti-EGFR groups (Figure 7a). While PLGA-PEG@DOX/anti-EGFR was also able to diffuse into tumors to some extent, it did so to a lesser extent in the absence of UMC treatment as compared to the PLGA-PEG@DOX/anti-EGFR + UMC group. This may be attributable to temporary UMC-induced changes in the interstitial fluid pressure within tumors, thus supporting more robust drug diffusion therein [35]. Subsequent H&E and TUNEL staining of these tumor-tissue samples revealed extensive cellular damage within tumors in the PLGA-PEG@DOX/anti-EGFR + UMC treatment group (Figure 7b), consistent with extensive tumor-cell destruction. In TUNEL staining images, dark brown nuclei were indicative of apoptotic cell death (Figure 7b), consistent with the above staining results. Together, these results confirmed that PLGA-PEG@DOX/anti-EGFR + UMC treatment was detrimental to TNBC tumor growth primarily by promoting intra-tumoral DOX penetration. 

## 4. Conclusions

PLGA-PEG@DOX/anti-EGFR nano-micelles were used as a therapeutic tool for the treatment of TNBC in combination with UMC. This analysis revealed that EGFR-based active targeting was effective in ensuring an enhanced DOX delivery to tumor sites, thereby augmenting the antitumor efficacy of this chemotherapeutic drug while decreasing its off-target toxicity. The use of SonoVue^TM^ as a cavitation nucleus in combination with US exposure at a power level of 0.5 W/cm^2^ markedly enhanced blood perfusion within tumors, maintaining high levels of viability. Relative to PLGA-PEG@DOX/anti-EGFR treatment alone, a combination of these PLGA-PEG@DOX/anti-EGFR nano-micelles and UMC was able to inhibit TNBC tumor growth even more effectively at lower concentrations. Importantly, UMC was found to be both safe and effective as a means of enhancing the intra-tumoral delivery of these DOX-loaded nano-micelles. Even so, this study is limited by the relatively short treatment duration and small-animal model systems utilized. Future research regarding the use of UMC in the context of chemotherapy will be necessary to fully explore the value of this treatment strategy in the clinical treatment of patients with TNBC and other cancer types. 

## Data Availability

The data presented in this study are available on request from the corresponding author. The data are not publicly available due to privacy and ethical reasons.

## Supplementary Materials

The following are available online at https://www.mdpi.com/article/10.3390/cancers13143383/s1, Figure S1: TEM images of PLGA-PEG@DOX nano-micelles; Figure S2: TEM images of PLGA-PEG@DOX/anti-EGFR nano-micelles stability in cell-culture medium at 37 °C for 3 days; Figure S3: Standard curve of the DOX concentration; Figure S4: The viability of MDA-MB-468 cells after US irradiation (0–1.5 W/cm^2^) in 5 min; Figure S5: Cellular uptake of DOX-loading nano-micelles by MCF-7 and MDA-MB-468 cells using flow cytometry; Figure S6: Body weight changes during the 21 days with or without PLGA-PEG/anti-EGFR treatment; Figure S7: Blood routine with or without PLGA-PEG/anti-EGFR nano-micelles for 21 days; Figure S8: Liver and kidney function treated with or without PLGA-PEG/anti-EGFR nano-micelles for 21 days; Figure S9: Histological analysis of the main organs of untreated mice (PBS) and mice treated with PLGA-PEG/anti-EGFR nano-micelles for 21 days; Figure S10: Body weight changes during the 15-d treatment observation period.
