# Supplementary material for: Ultrasound-Mediated Cavitation Enhances EGFR-Targeting PLGA-PEG Nano-Micelle Delivery for Triple-Negative Breast Cancer Treatment
Source: Cancers (Basel). 2021 Jul 6;13(14):3383. doi: 10.3390/cancers13143383 (PMC8304156; doi:10.3390/cancers13143383)
Supplement: Supplementary file 1 [file cancers-13-03383-s001.zip › cancers-1269200-SI.pdf]

cancers

MDPI

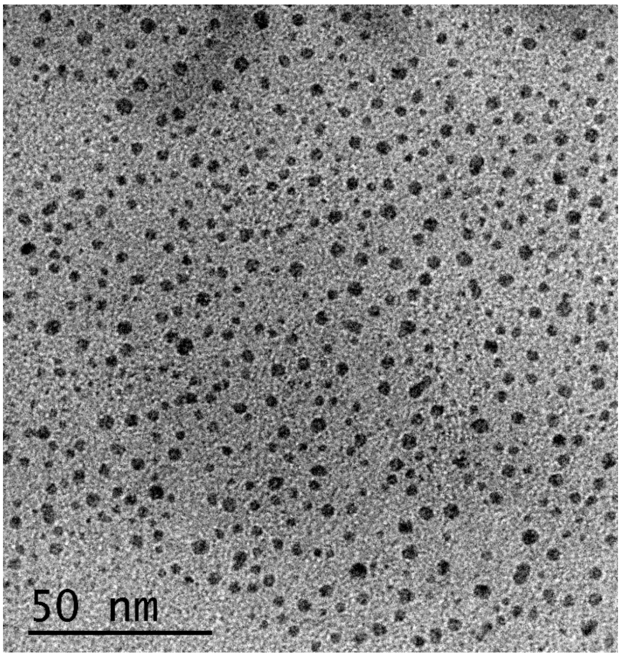

Figure S1. TEM images of PLGA-PEG@DOX nano-micelles. Scale bar: 50 nm.

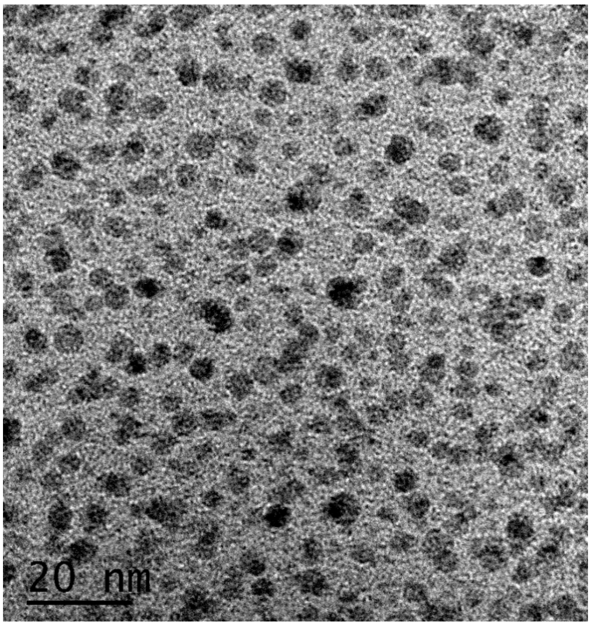

Figure S2. TEM images of PLGA-PEG@DOX/anti-EGFR nano-micelles stability placed in cell culture medium at 37 °C for 3 days. Scale bar: 20 nm.

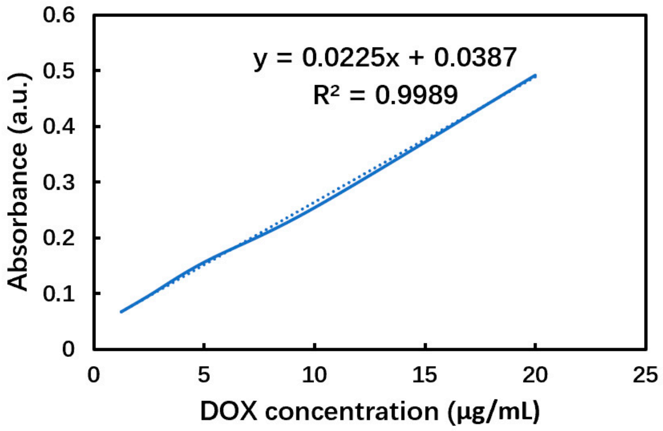

Figure S3. Standard curve of the DOX concentration.

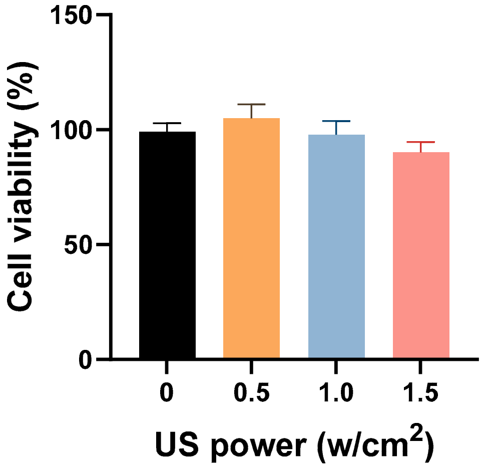

Figure S4. The viability of MDA-MB-468 cells after 5 min of US irradiation (0-1.5 W/cm2) in 5 min.

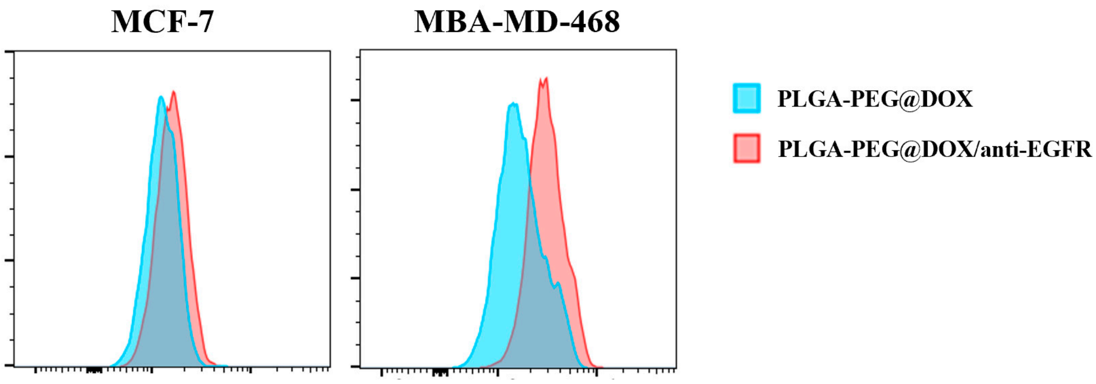

Figure S5: Cellular uptake of DOX-loading nano-micelles by MCF-7 and MDA-MB-468 cells using flow cytometry.

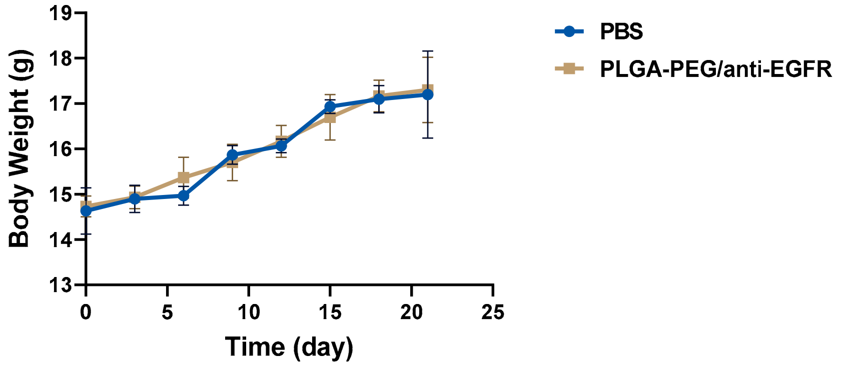

Figure S6. Body weight changes over time with or without PLGA-PEG/anti-EGFR nano-micelles (n = 3).

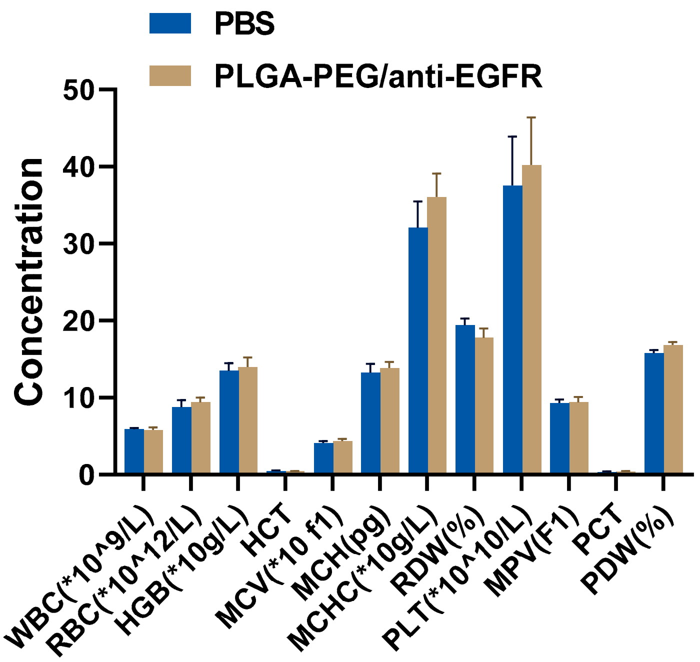

Figure S7. Blood routine, indicators include white blood cells (WBC), red blood cells (RBC), hemoglobin (HGB), hematocrit (HCT), mean corpuscular volume (MCV), mean corpuscular hemoglobin (MCH), mean corpuscular hemoglobin to determine blood drug concentration (MCHC), red blood cell distribution width (RDW), platelets (PLT), mean platelet volume (MPV), and platelet distribution width (PDW) with or without PLGA-PEG/anti-EGFR nano-micelles for 21 days (n=3).

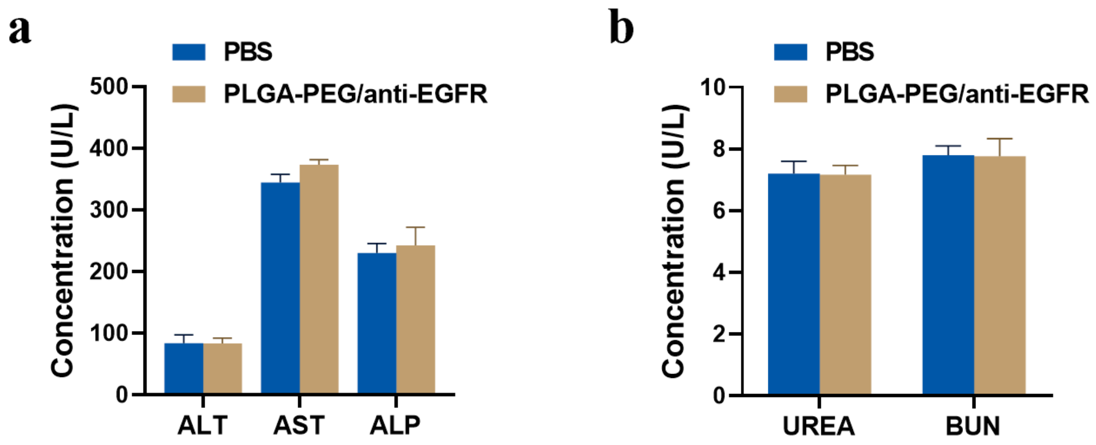

Figure S8. (**a**) Liver function markers, including alanine aminotransferase (ALT), aspartate aminotransferase (AST), and alkaline phosphatase (ALP), as well as (**b**) renal function markers including serum urea (UREA) and blood urea nitrogen (BUN) with or without PLGA-PEG/anti-EGFR nano-micelles for 21 days (*n*=3).

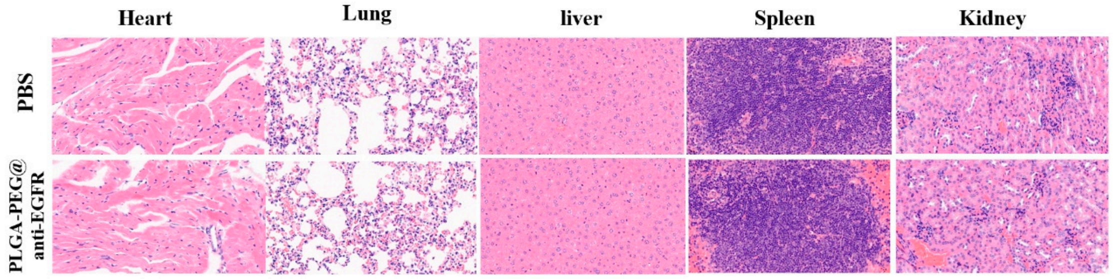

Figure S9. Histological analysis of the main organs (heart, liver, spleen, lung and kidney) of untreated mice (PBS) and mice treated with PLGA-PEG/anti-EGFR nano-micelles for 21 days.

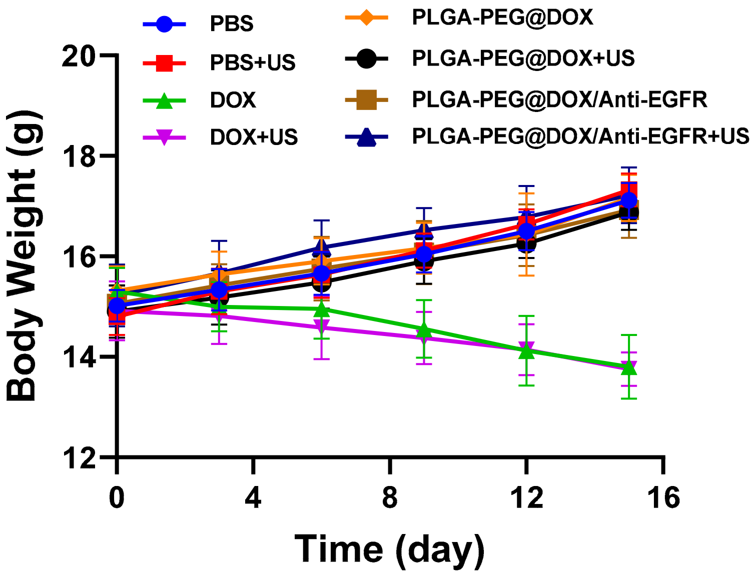

Figure S10. Body weight changes during the 15-day treatment observation period (n = 5).
